# Geocoding cryptosporidiosis cases in Ireland (2008–2017)—development of a reliable, reproducible, multiphase geocoding methodology

**DOI:** 10.1007/s11845-020-02468-0

**Published:** 2021-01-19

**Authors:** Lisa Domegan, Patricia Garvey, Paul McKeown, Howard Johnson, Paul Hynds, Jean O’Dwyer, Coilín ÓhAiseadha

**Affiliations:** 1 European Programme for Intervention Epidemiology Training (EPIET), European Centre for Disease Prevention and Control (ECDC), Stockholm, Sweden; 2 Health Service Executive-Health Protection Surveillance Centre, Dublin, Ireland; 3 Health Service Executive-Health Intelligence Unit, Dublin, Ireland; 4 Environmental Sustainability & Health Institute, Technological University Dublin, Dublin, Ireland; 5 Irish Centre for Research in Applied Geosciences, University College Dublin, Dublin, Ireland; 6 School of Biological, Earth and Environmental Sciences, University College Cork, Cork, Ireland; 7 Water and Environment Research Group, Environmental Research Institute, University College Cork, Cork, Ireland; 8 Health Service Executive-Department of Public Health-East, Dublin, Ireland

**Keywords:** Geocoding, Geospatial, Epidemiology, Data quality, Surveillance, Cryptosporidiosis

## Abstract

**Background:**

Geocoding (the process of converting a text address into spatial data) quality may affect geospatial epidemiological study findings. No national standards for best geocoding practice exist in Ireland. Irish postcodes (Eircodes) are not routinely recorded for infectious disease notifications and > 35% of dwellings have non-unique addresses. This may result in incomplete geocoding and introduce systematic errors into studies.

**Aims:**

This study aimed to develop a reliable and reproducible methodology to geocode cryptosporidiosis notifications to fine-resolution spatial units (Census 2016 Small Areas), to enhance data validity and completeness, thus improving geospatial epidemiological studies.

**Methods:**

A protocol was devised to utilise geocoding tools developed by the Health Service Executive’s Health Intelligence Unit. Geocoding employed finite-string automated and manual matching, undertaken sequentially in three additive phases. The protocol was applied to a cryptosporidiosis notification dataset (2008–2017) from Ireland’s Computerised Infectious Disease Reporting System. Outputs were validated against devised criteria.

**Results:**

Overall, 92.1% (4266/4633) of cases were successfully geocoded to one Small Area, and 95.5% (*n* = 4425) to larger spatial units. The proportion of records geocoded increased by 14% using the multiphase approach, with 5% of records re-assigned to a different spatial unit.

**Conclusions:**

The developed multiphase protocol improved the completeness and validity of geocoding, thus increasing the power of subsequent studies. The authors recommend capturing Eircodes ideally using application programming interface for infectious disease or other health-related datasets, for more efficient and reliable geocoding. Where Eircodes are not recorded/available, for best geocoding practice, we recommend this (or a similar) quality driven protocol.

## Background

Significant potential exists for improving our understanding of the epidemiological mechanisms (e.g. source attribution) of infectious diseases by investigating associations between disease incidence and key environmental, infrastructural and meteorological drivers/risk factors. Geospatial epidemiological studies may be undertaken across a range of spatial units (from Census Small Areas to national and multi-country regions) and over long study periods (from weeks to decades), and may include multiple levels of risk factor interactions (e.g. population age structure, socioeconomic status, meteorological events, metagenomics) in addition to multiple thousands of cases. In this era of ‘Big Data’ and particularly in light of the COVID-19 pandemic [1, 2], new and increasingly technical approaches to disease mapping and data integration in Ireland are required.

A key first step in retrospective geospatial epidemiological investigations is geocoding of infectious disease datasets, thus enabling spatially specific linkage with existing secondary datasets. Geocoding is the computational process of transforming a physical address description to a spatially useful and comparable representation (e.g. geographical coordinates) of that location [3]. The quality of geocoded data may be assessed in terms of the following four criteria: completeness, positional accuracy, concordance with correct spatial units and repeatability [4–6]. The quality of input address data (in terms of completeness, spellings, and correct address components) impacts on the completeness and validity of geocoding [7, 8].

Several previous studies have applied geocoding to gastroenteric infection datasets in order to conduct ecological studies of cryptosporidiosis, verotoxigenic *Escherichia coli* (VTEC) enteritis and other infectious intestinal diseases [9–12]. For example, a study by ÓhAiseadha et al. (2017) employed high-resolution geocoding to construct an anonymised dataset of VTEC infections in Ireland, thus permitting geo-linking of agricultural and infrastructural risk factors and sociodemographic drivers, with human health outcomes. ÓhAiseadha et al. geocoded VTEC records using Ireland’s Health Atlas Geo Reference platform, using a two-step process comprising automated and manual address matching. This study was the first from Ireland to report a marked rural-urban inequality in the cumulative incidence of disease, identifying cattle density and private well reliance as geospatial determinants of VTEC infection [9]. Similarly, Pollack et al. investigated the association between *Cryptosporidium parvum* infections and rurality, characterised by higher ratios of farms to human inhabitants and of private water supplies to human inhabitants [10]. Pollack et al. geocoded all individual cryptosporidiosis records to a postcode sector based on the reported postcode, with cases occurring within a postcode sector spatially referenced to the postcode centroid (i.e. latitude-longitude).

*C. parvum* is a protozoan parasite of ruminant animals with incidental human infection typically associated with farming activities or direct animal contact. Epidemiological studies have revealed that exposure to drinking water from private and public supplies represents an increased risk for cryptosporidiosis [13, 14]. In Ireland, cases of cryptosporidiosis are commonly associated with *C. parvum* and predominantly reported from rural areas [14, 15]. The crude incidence rate (CIR) of cryptosporidiosis in Ireland has been slowly increasing in recent years, ranging from 8.6/100,000 in 2014 to 13.2/100,000 in 2018 [9], with the highest CIRs reported in Europe [16]. Studies are thus required to increase our scientific understanding of the associations between cryptosporidiosis occurrence in Ireland and key environmental, infrastructural and meteorological drivers/risk factors and to translate these findings into geospatially targeted environmental, infrastructural and health policies and public health interventions.

For geospatial epidemiological studies based on area of residence, there are a number of limitations with Irish addresses. For example, over 600,000 (35%) Irish premises have shared non-unique addresses (i.e. addresses shared by more than one property/dwelling) due to an absence of house numbers or names. This occurs predominantly in rural areas, where approximately 37.3% of the Irish population resides [17]. Townlands continue to be used as a primary address descriptor in Ireland, particularly in rural areas. A townland is a geographical division of land; the Central Statistics Office (CSO) listed 50,117 townlands in Ireland at the time of Census 2016 [18].

In 2015, each residential and business address in Ireland was assigned a unique address postcode, known as an Eircode [19]. Ireland was the last country in the OECD to create a comprehensive national postcode system. However, Eircodes are not routinely recorded for infectious disease notification surveillance.

Infectious diseases with higher incidence rates in rural areas (e.g. cryptosporidiosis), may be characterised by lower level of efficiency and validity during the geocoding process, potentially introducing biases into geospatial epidemiological studies. In the study by ÓhAiseadha et al. (2017), 80% of confirmed index cases of VTEC enterocolitis were geocoded to one Census 2011 Small Area (SA); the remaining 20% of ambiguous (or invalid) addresses were left uncoded to maximise validity, albeit with some loss of statistical power, while also potentially introducing some level of bias into the study. There is a pressing need for a standardised protocol for geocoding infectious disease notification data in Ireland, to improve the validity and reliability of geocoding and hence data quality of geocoded events. Moreover, application of a systematic approach to geocoding improves transparency of study outputs and reproducibility for future studies, while also reducing ‘data loss’.

The overall aim of this paper was to describe the development of a reliable, reproducible protocol to geocode spatiotemporal infectious disease datasets for use in geospatial epidemiological studies, in order to improve data validity, maximise data completeness and identify possible biases.

A 10-year (2008–2017) national dataset comprising address-level cryptosporidiosis notifications from Ireland’s Computerised Infectious Disease Reporting System (CIDR) is used to present the developed protocol.

## Methods

Study objectives:

1. Develop a protocol to geocode infectious disease notifications, using notified cryptosporidiosis case data;
2. Evaluate the reliability of the developed protocol;
3. Produce a geocoded spatiotemporal dataset comprising notified infectious disease cases, with each case linked to one Census 2016 Small Area (SA), or larger spatial unit.

### CSO Census Small Areas

A dataset comprising notified cryptosporidiosis cases was geocoded, with each case linked to one CSO Census 2016 Small Area (SA), or larger spatial unit. SAs are delineated spatial areas, generally comprising between 80 and 120 dwellings (mean: 90) and represent the smallest geographical unit (i.e. the unit of highest spatial resolution) available for compilation/reporting of statistics in line with data protection regulations. They generally comprise either complete or parts of townlands, or neighbourhoods. SAs nest within Electoral Division (ED) boundaries, which are the smallest legally defined administrative areas in Ireland. EDs are recognised by the European Union as ‘local administrative units (LAU) Level 2’. As of 2016, there were 18,641 SAs and 3440 EDs delineated in Ireland for the national Census [20, 21].

### Ethical approval, data protection and data sources

Following receipt of ethical approval for this study from the Royal College of Physicians of Ireland Research Ethics Committee (RECSAF_84), access to address-level infectious disease notification data from Ireland’s CIDR system [22] at the national level was sought and subsequently granted by the National CIDR Peer Review Group. For data protection purposes, aside from address information, personal data and disease-specific information were excluded from the datasets during the geocoding process. Datasets for geocoding were only accessed by designated partners working within the HSE, including the Heath Protection Surveillance Centre (HSE-HPSC), the Department of Public Health-East and the Health Intelligence Unit (HIU). Data security and confidentiality were maintained at all times, in compliance with the requirements of data protection legislation, specifically the General Data Protection Regulation (GDPR) and the Data Protection Act 2018. HSE-HPSC is accredited for Information Security Management ISO 27001.

CIDR is an information system developed to manage the surveillance and control of infectious diseases in Ireland, using standard case definitions for all notifiable diseases, as per the Infectious Diseases (Amendment) Regulations 2020 (S.I. No. 53 of 2020) [22]. The cryptosporidiosis dataset comprised a 10-year (2008–2017) dataset including address-level infectious disease notification national data from CIDR. The variables used for geocoding included a unique identifier (CIDR Event ID) and the spatial identifier variables: Address Line 1, Address Line 2, Town, Suburb, Postcode and County.

### Geocoding

This protocol employs the following: (i) a Health Intelligence Unit (HIU) in-house geocoding program and (ii) the Geo Reference tools on the Health Atlas Ireland platform. Health Atlas Ireland comprises a suite of software tools developed more recently by the HIU to provide role-based web access to key health-related datasets and is limited to HSE and partner organisations [23–25]. Geo Reference, one of the software applications accessed via Health Atlas Ireland, uses An Post’s GeoDirectory for the purposes of geocoding, thus facilitating mapping and geospatial analyses. The GeoDirectory is a definitive reference dictionary of addresses for all 1.9 million buildings that receive post in the Republic of Ireland, assigning them with precise postal and geographic addresses. Irish postcodes (called ‘Eircodes’) are generated by the company Eircode and licensed to other parties [19], including GeoDirectory.

**Fig. 1:**
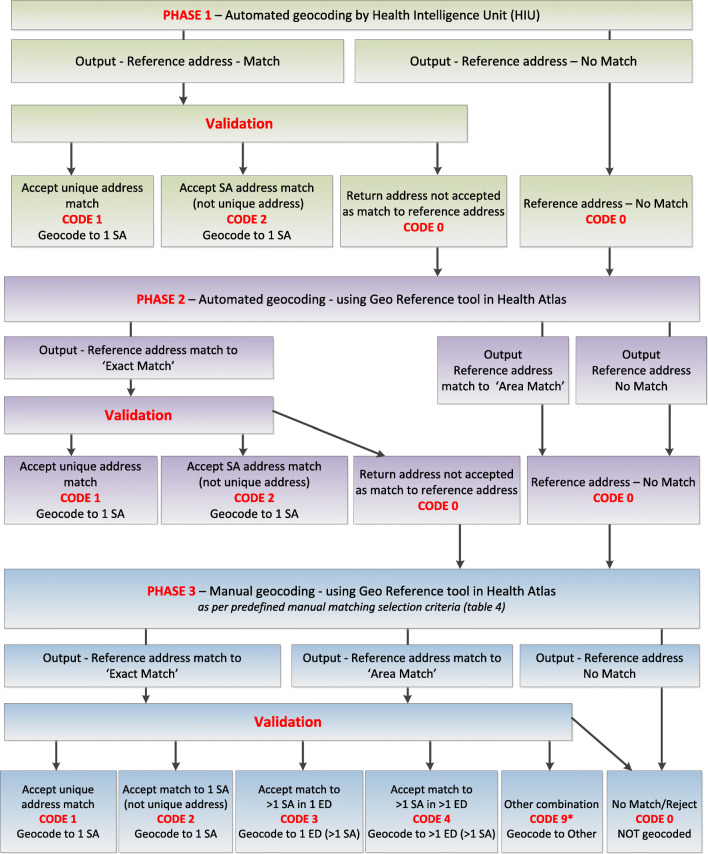
Decision tree outlining the developed geocoding protocol, delineated by distinct methodologies employed (Phases 1–3). The ‘Match Type Code’ (0–9) is defined in Table 1

Geocoding of residential addresses associated with cryptosporidiosis notification data involved a series of string-matching algorithms i.e. exact string and finite string matching, undertaken sequentially in three phases (Phases 1–3); two automated phases and one manual phase (Fig. 1). In this study, the address of a cryptosporidiosis case reported in the CIDR dataset is referred to as the ‘reference address’. All address fields (Address Lines 1 & 2, Town, Suburb, Postcode and County) in the CIDR dataset were included in the geocoding process. Each record was manually assigned a numeric match type code, defining the type of spatial unit the address was geocoded to (Table 1).

**Table 1:** Definitions for generated variable ‘Match Type Code’ (SA, small area; ED, electoral division)

Match type code	Description
0	Records not geocoded, i.e. records not matched to XY coordinates/1 SA or other geographical unit; OR Records whose match was rejected during validation.
1	Records geocoded to a unique address (match level ‘XY’)
2	Records geocoded to 1 SA, but not to a unique address
3	Records geocoded to > 1 SA (in 1 ED)
4	Records geocoded to > 1 SA in > 1 ED
9	Records geocoded to ‘Other’ combinations of SAs and EDs.

### Geocoding Phase 1

An automated geocoding program attempts to match each address in the submitted CIDR dataset (i.e. the ‘reference address’) to an address from An Post’s GeoDirectory by searching for individual components (e.g. house number/name, apartment complex, street, townland, suburb, town, county) of the reference address among addresses in the GeoDirectory, using exact string matching. In the event of a likely address match identified by several identical or near-identical components, the program returns the XY geographical coordinates for the matched address (i.e. the ‘returned address’). For any address that remains unmatched (i.e. no return address identified), the program attempts to search again using synonyms from a pre-existing data frame of paired strings (e.g. George St Gt N-North Great George’s Street), using literal string matching. The algorithm is thus described as a ‘naive string search’ with ‘normalisation’ [26]. The program does not provide a measure of distance or proximity to each match.

A unique address match was considered to be achieved when a unique dwelling identifier in the returned address matched that in the reference address. A unique dwelling identifier refers to a house name, a house number in combination with a unique street name, an apartment number in combination with a unique apartment complex name, or a complete Eircode. Where the returned address was matched to SA level, the output was validated against a set of pre-defined validation criteria developed by two members of the research team (Table 2).

**Table 2:** Validation criteria for all output records with a reference address possible match to returned address from Phase 1 and 2 automated geocoding

Validation issue	Validation check(s)	Accept/reject
Minor differences between reference and returned address	Normalisation checks—punctuation and abbreviations	Accept
	Address order differences	Accept
	Irish/English language variations	Accept
	If house identifier and ≥ 2 other address components match those in reference address	Accept
Returned address includes additional information	If house identifier and ≥ 2 other address components match those in reference address	Accept
Returned address omits components of reference address	If house identifier and ≥ 2 other address components match those in reference address	Accept
Ambiguity at the fine level (urban addresses)	Returned address has different house/apartment number.	Accept—if near neighbour* (in same apartment complex/same street in small town). Reject if street traverses diverse terrain, e.g. urban area with dense housing, suburban area with parks and/or rural area with fields.
Ambiguity at the fine level (rural addresses)	Returned address offers different house name.	Accept if returned townland matches reference townland. Reject if returned townland does not match reference townland.
No dwelling identifier (rural addresses)	Returned address does not include a unique dwelling identifier.	Accept if returned townland matches reference townland.
No dwelling identifier (urban addresses)	Returned address does not include a unique dwelling identifier.	Accept if returned short street and small town match reference street and town. Reject if returned street is numbered to ≥ 100.
Unique dwelling identifier with address components missing/added	Returned address based on unique dwelling identifier. The returned address includes additional address components or omits address components, e.g. nearby crossroads named in reference address but not included in returned address, or townland not named in reference address but included in returned address.	Accept

An additional variable ‘Match Type Code’ was created in the dataset to code the address match level (Table 1). Addresses that met the validation criteria for a unique address match to XY coordinates were coded ‘1’ and records with a validated address match to one Small Area (SA), but not to a unique address, were coded ‘2’. Records that remained unmatched after Phase 1, including those that were matched but did not pass validation, were coded ‘0’ (Fig. 1). The objectives of the validation criteria and the match type coding were to improve the quality of the geocoding process via development of a series of ‘data-bins’ i.e. grouping of records based on the level of geocoding validity and reliability. The match type code also served as a measure of confidence in the match.

Once matching was complete, each address was spatially attributed via its geographical coordinates to the corresponding SA, i.e. reverse-geocoded from a point to a geographical unit that was specifically developed for population analyses. Reverse geocoding to a geographical centroid of SA i.e. latitude/longitude, maintains spatial accuracy, while also safeguarding anonymity.

### Geocoding Phase 2

All records with no address match (coded ‘0’) and records that failed the validation process during Phase 1 (also coded ‘0’) were included in the second geocoding phase (Phase 2; Fig. 1). These records were uploaded to the Health Atlas Geo Reference tool for automated address matching using an approximate (‘fuzzy’) string-matching algorithm [27]. Approximate string-matching attempts to match components of the reference address which were identified in the GeoDirectory. Further matches are possible by allowing for erroneous characters (e.g. ‘Ballyboug’ matched to ‘Ballybough’) or typographical transpositions (e.g. ‘Dulbin’ for ‘Dublin’).

All output records with a reference address ‘exact match’ (i.e. a unique address match) to a return address from Phase 2 of geocoding were validated against pre-agreed validation criteria analogous to those applied during Phase 1 validation (Table 2). All outputs with a reference address ‘area match’ (i.e. to a cluster of addresses) or with ‘no match’ from Phase 2 and all validated records not accepted as a match to a return address were coded ‘0’. These records underwent a third, manual geocoding step (Phase 3; Fig. 1).

### Geocoding Phase 3

To maximise the number of records successfully geocoded, a manual geocoding process was applied. A ‘fuzzy search’ function in the Health Atlas Geo Reference tool was used to identify further appropriate matches in the GeoDirectory database (via approximate string matching, with multiple returned approximations) for the remaining unmatched records. The fuzzy search function automatically returns a list of all addresses that are approximately matched to each reference address, permitting the user to manually match addresses that may contain variant spellings, misspellings, typographical errors or Irish-language equivalents not identified during automated Phases 1 and 2. Criteria were devised for manual matching addresses to SA/ED using the fuzzy search function (Table 3). These criteria were devised for geocoding to SA, ED or larger spatial units; i.e. a unique address match was not required.

**Table 3:** Manual matching criteria for selection of geocoded address matches from options produced by the Health Atlas Geo Reference tool’s fuzzy search function (*A ‘similar’ dwelling name refers to minor punctuation/spelling differences in the house name)

Address type	Fuzzy search output	Matching decision	Spatial unit of match level
Rural	Of returned address(es), there is one address with dwelling namesame/similar* to reference address. Other address components (townland,town and county) match the reference address.	Unique address match	1 SA (in 1 ED)
	Multiple addresses are returned as potential matches to the reference address.None of the returned addresses lists the same/similar house name. Ondeletion of the house name, several of the returned addresses list thesame townland and county as in the reference address.	Area match to all returnedaddresses listing the sametownland and county	1 SA (in 1 ED) **OR** > 1 SA (in 1 ED) **OR** > 1 SA (in > 1 ED)
Urban	Of returned addresses, one address has the same house/apartment numberas in the reference address, or the same/similar* house name AND • All other address components (street, town/city and county) match thosein the reference address OR • Street, suburb and county match those in reference address OR • Street and county match those in the reference address. The returnedaddress identifies an adjacent suburb OR • The address identifies the same street and the same Dublin postcode(or same first part of Eircode) as the reference address.	Unique address match	1 SA (in 1 ED)
	Multiple addresses listing numbered/named houses or apartments arereturned as potential matches to the reference address. None of thereturned addresses has the same dwelling number/name as thereference address AND • All other address components (street, town/city and county) match thosein the reference address OR • Street, suburb and county match those in the reference address OR • Street and county match those in the reference address. The returnedaddress identifies an adjacent suburb OR • Street matches that in the reference address. The address identifies sameDublin postcode or same first part of Eircode as in the reference address.	Match to nearestnumbered/named dwellingof returned addresses in thesame SA	1 SA (in 1 ED) **OR** > 1 SA (in 1 ED) **OR** > 1 SA (in > 1 ED)
	Of returned addresses, no address has the same/similar dwellingname/number, street and suburb. On deleting the suburb from thereference address, one of the returned addresses has the same/similardwelling name/number, same street and adjacent suburb.	Unique address match	1 SA (in 1 ED)
Urban or rural	Multiple addresses are returned as potential matches to the referenceaddress. Of returned addresses, several addresses have • The same/similar dwelling name AND • Same street name AND • Different Dublin postcode/first part of Eircode.	Area match	County
	• Multiple addresses are returned as potential matches to the referenceaddress. Several addresses with dwelling name same/similar toreference address, but townland differs OR • No addresses returned in Health Atlas Geo Reference OR • Selection criteria for manual matching cannot be met, due to multipleaddress components missing in reference address (e.g. no addressdata except County) OR • Reference address in another country, e.g. England	Not matched	Not geocoded

In conjunction with the selection criteria for manual matching, the Health Atlas Geo Reference ‘Area match’ function was used to check whether any given group of addresses, selected from the GeoDirectory, would successfully match to 1, 2 or 3 Small Areas before proceeding to ‘Save as area match’. The ‘Display on map’ function provided an additional visual tool to inspect if a specific cohort of addresses are situated within a well-defined geographical area, e.g. a single townland, before proceeding with the area match.

### Validation, identification of potential bias and quality control

Records that could not be geocoded, records that failed validation during each phase and records geocoded in each phase were reviewed in order to identify any potential spatiotemporal bias that may impact on future studies/sensitivity analyses by inclusion/exclusion of these records. Records that failed validation are those with a potential address match that did not meet the outlined validation criteria/manual matching criteria.

A number of quality control procedures were implemented to improve the overall efficacy of geocoding, outlined as follows:

- All criteria for geocoding and validation were devised and revised through an extensive iterative process, involving members of the research team. Ambiguous addresses that did not match the initial criteria for matching were assessed with a view to devising appropriate new validation criteria;
- The final validation criteria were reviewed and agreed by the whole research team;
- A variable recording the type of geocoding match, e.g. a unique address match or match to one SA, was added for each record to allow the flexibility to conduct sensitivity analysis during subsequent epidemiological studies if required;
- The Geo Reference validation tools (Area match and Display on map functions) were used to verify and maximise matching;
- Validation/verification of townlands was conducted using the Irish townlands database. https://www.townlands.ie/

## Results

### Geocoding output

A total of 3740/4633 (80.7%) addresses of laboratory-confirmed cryptosporidiosis cases (records) were successfully geocoded to one SA during Phase 1 of the developed geocoding protocol, of which 67.1% (2511/3740) of records were matched to a unique address. A further 1611 records were initially matched to a unique SA, of which 1229 met the validation criteria. Following phase 1, 8.2% (382/4633) of records failed validation and a further 11% (511/4633) remained unmatched by the exact-matching algorithm.

During the second phase of automated geocoding, of 893 (which included 382 records that failed validation and 511 unmatched records) remaining records, 70/893 (7.8%) were geocoded to one SA. Following completion of geocoding from Phases 1 and 2, 3810/4633 (82.2%) of records were geocoded to one SA. Of the remaining records (*n* = 823), an additional 456/823 (55.4%) records were geocoded by manual address matching to one SA (Table 3).

**Fig. 2:**
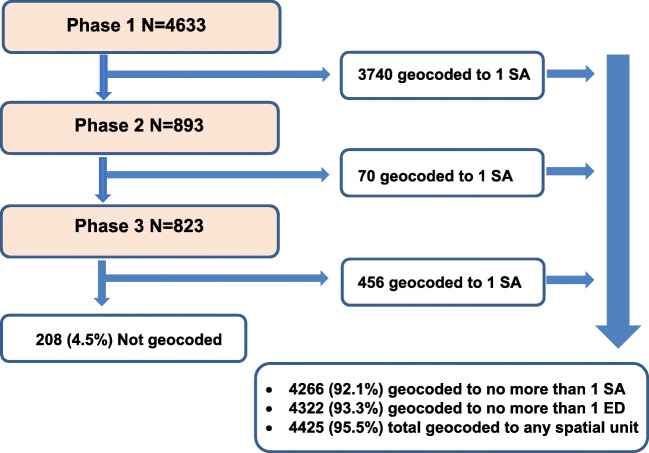
Flowchart of output from three geocoding phases, including number of records geocoded to one Small Area during each phase and the total number of records geocoded and not geocoded following completion of the multiphase methodology

Following the three phases of geocoding and validation, 92.1% (4266/4633) of cryptosporidiosis records were successfully geocoded to one SA and 93.3% (4322/4633) were geocoded to one ED. In total, 95.5% (4425/4633) of all records were geocoded to a geographical area (including areas > 1 ED) (Fig. 2, Table 4). During Phases 2 and 3, an additional 526 records were geocoded to one SA, increasing the total number of records geocoded to one SA by 14.1% (526/3740).

**Table 4:** Number and percentage of all cryptosporidiosis records geocoded following completion of the geocoding process (Phases 1–3), N = 4633

		Phase 1		Phase 2		Phase 3		Phases 1–3	
	*Spatial unit*	Number	%	Number	%	Number	%	Number	%
Total records	**-**	4633	100	893	100	823	100	4633	100
Match to a unique address	XY coordinate	2511	54.2	59	6.6	191	23.2	2761	59.6
Match to only 1 SA (not to a unique address)	SA	1229	26.5	11	1.2	265	32.2	1505	32.5
Match to 1 SA—failed validation	SA	382	8.2	35	3.9	54	6.6		
No match to reference address	-	511	11.0	788	88.2	154	18.7		
Geocoded to only 1 SA^a^	SA	3740	80.7	70	7.8	456	55.4	4266	92.1
Geocoded to only 1 ED (≥ 1 SA)	ED	-	-	-	-	512	62.2	4322	93.3
Geocoded to any spatial unit	All	-	-	-	-	615	74.7	4425	95.5
Records not geocoded	-	-	-	-	-	-	-	208	4.5

^a^Including records geocoded to a unique address

### Validation output

Frequently observed address validation issues were the use of non-unique townland addresses, spelling variants (including the Irish language) and incomplete/partial addresses.

Records with a possible match to a return address following each geocoding phase were validated against a set of devised validation criteria. Of records geocoded to one SA, 9.3% (382/4122) failed the validation process during Phase 1 of geocoding, 33.3% (35/105) failed validation in Phase 2 and 10.6% (54/510) failed validation in Phase 3.

Of 4122 records geocoded to one SA during the first phase, 5.4% (223/4122) were incorrectly assigned to a spatial unit. Of these 223 records, 137 were re-assigned to a different SA and 86 records to a larger spatial unit (i.e. > 1 SA) during phase 3 manual geocoding phase.

Of the 208 records not successfully geocoded after all three phases, 70% were notified before 2012. Regional differences were observed, with 49% of records that could not be geocoded deriving from one HSE area. Regional differences were also observed with the additional 526 records geocoded to one SA during phases 2 and 3, 72% of the records were notified from four HSE-Areas (Mid-West, South, South-East and West) and 25% of records were notified from only two counties—Cork and Galway.

## Discussion

We have developed a reliable, reproducible protocol for geocoding Irish health-related datasets for subsequent use in geospatial epidemiological studies. Over 92% (*n* = 4266) of cryptosporidiosis records were successfully geocoded to one SA and 96% (*n* = 4425) to any spatial unit. The developed multiphase geocoding protocol was found to improve overall data completeness of geocoded records to one SA by 14.1% between the first and final phase of geocoding (i.e. from 81% in phase 1, to 92% following all phases—an additional 526 cases). The validation process has ensured a high level of data quality, with 5% of records re-assigned to a different or larger spatial unit (i.e. > 1 SA) following validation. For studies involving multiple thousands of records, the overall improvement in geocoding completeness (by 14%) and concordance (with 5% records re-assigned to the correct spatial unit) may significantly impact study findings.

During development of this geocoding protocol, the research team compared the level of geocoding quality comparing single-phase geocoding to a multiphase approach. Data quality issues in geocoded datasets may include incompleteness, positional errors and incorrect assignment to geographic units, which may introduce substantial bias in spatial analysis [28]. Increasing the number of records geocoded for inclusion in geospatial epidemiological studies will result in better models for mechanistic understanding and eventual mitigation, with the degree of potential bias limited by the high level of completeness achieved. Previous studies have shown that 20–26% of records may be excluded from studies due to incomplete geocoding to fine-resolution spatial units [4, 9]. In this study, > 92% of records were geocoded to one SA and the level of spatial unit that each record was assigned to was defined for all records, thus enabling further assessment of potential bias based on the level and quality of geocoding.

The rationale for applying two automated matching algorithms (during Phase 1 and 2) in sequence, was justified, as the second algorithm successfully identified additional matches, that had not been not identified by the first exact-string algorithm (*n* = 70). Geocoding methodologies (single-phase versus a multiphase approach), can be sensitive to sparsely populated rural areas [29], possibly due to incomplete address-level information in rural areas. The number of records successfully geocoded was maximised by applying a third manual geocoding process, Phase 3, using approximate string matching, alongside manual pre-processing and validation. The application of a manual geocoding phase resulted in improved geocoding completeness and higher confidence in geocoding concordance, consistent with the findings of previous authors that manual geocoding correction is ‘both a feasible and economical method for improving the quality of geocoded data’ [7]. The quality and speed of manual geocoding can however depend on the experience of the geocoder, which further highlights the need for a national geocoding protocol.

The 10-year cryptosporidiosis notification dataset from Ireland’s national surveillance and notification system (CIDR) resulted in fewer than 5% (208/4633) of notified cases that could not be reliably geocoded to a spatial unit, due to insufficient address-level information. Most of these records were notified before 2012, with a significant proportion notified from one particular HSE area. The phased national implementation of CIDR by regions over time up to 2012 accounted for some of these regional differences. In addition, one-third of all records that could not be geocoded were notified during the 2009 influenza pandemic period. During this time, the impact of the 2009 influenza pandemic meant that surveillance and resources were, of necessity, redirected, and this may have affected the validation of infectious disease notifications. Of all cryptosporidiosis notifications (2008–2017), data quality issues with regard to address-level information were identified in just 1% of records after 2011. Regional differences were also observed in the records geocoded to one SA during phases 2 and 3. This identification of non-random differential completeness/validity of address variables may result in potential spatiotemporal bias and cartographic confounding [4], particularly for geospatial studies focused on rural/urban differences. The creation of ‘data bins’ in this study, will facilitate future sensitivity analyses, that can be conducted on cryptosporidiosis datasets with varying inclusion/exclusion criteria, based on the validity of the geocode and the assigned spatial unit.

We explored the issues that arise in geocoding health data in Ireland—where Eircodes are not routinely gathered and non-unique addresses plus quality issues abound. The most frequently observed address data quality issues included the use of non-unique townland addresses, ambiguity regarding house names, spelling variants (including the Irish language) and incomplete/partial addresses.

## Conclusion

The developed multiphase protocol achieved an overall geocoding success rate of 92% to one SA and 93% to one ED, indicating the significant potential of Irish infectious disease data for spatiotemporal investigation. Multiphase geocoding to SAs, EDs and larger geographic areas will enable greater flexibility with geospatial epidemiological studies, including sensitivity analyses. Manual validation confirmed that this multiphase geocoding methodology is reliable and the devised protocol ensures that it is reproducible. By improving the completeness and validity of geocoding processes using this protocol, the research team has produced a geospatial dataset that offers increased power and has identified potential biases, important for the design and analysis of subsequent geospatial epidemiological studies. The optimisation of geocoding completeness and validity is a prerequisite for enhanced surveillance of infectious diseases and plays a significant role in identifying relationships between environmental exposures and public health outcomes. The likelihood of detecting a genuine relationship between any environmental exposures and health outcomes depends not only on the strength of the relationship but also on geocoding quality [4–6].

The overall aim of this paper was to describe the development of a reliable, reproducible protocol to geocode spatiotemporal infectious disease datasets for use in geospatial epidemiological retrospective studies. However, aspects of the devised methodology can also be applied to real-time public health investigations from a live dataset such as COVID-19, in particular the geocoding methodology described for Phases 2 and 3. The validation rules and address matching criteria would facilitate real-time geocoding of records with address issues such as non-unique townland addresses, spelling variants (including the Irish language) and incomplete/partial addresses. The step by step multi-phase methodology presented in this paper would further improve data completeness and standardisation of geocoding COVID-19 and other infectious diseases/health related datasets. The protocol has any number of applications to health outcome datasets that include address data, enabling researchers to seek correlations between environmental and social determinants of population health. As an outcome of this research, the Health Atlas Ireland geocoding tool was enhanced to exploit Eircodes when present and to better display addresses for faster manual interpretation. Such systematisation underpins COVID-19 mapping in Ireland, including the GeoHive local electoral area presentation.

The COVID-19 pandemic has highlighted the urgent need for detailed geospatial epidemiological surveillance, mapping and research in Ireland, and globally [2]. This study has identified non-random differential completeness/quality of address level data during the previous 2009 pandemic. There is therefore a need to develop national standards for best geocoding practice for COVID-19 and other infectious disease epidemiological studies in Ireland. In the absence of such standards, we recommend following this multiphase methodology (or similar) as a national standard, to maximise the completeness and validity of geospatial data.

## Recommendations

The authors strongly recommend that, in future, Eircodes be captured for all infectious disease notifications and other health-related data in Ireland. Reporting Eircodes would improve the quality, standardisation and reliability of address-level information on CIDR (and subsequent geocoding), in line with recent Health Information and Quality Authority (HIQA) recommendations to improve data quality at all levels for CIDR [30].

The use of Eircodes combined with application programming interface (API) technology on CIDR/any future national notification systems will alleviate potential data quality issues that may arise with the use of postcodes alone [28]. By facilitating automated address matching and reducing uncertainty in validation, use of Eircodes combined with API would ultimately lead to a more efficient and less resource-intensive geocoding process.

For infectious disease notifications (with/without Eircodes recorded), data entry of address-level data on CIDR should be standardised and improved.

The protocol outlined in this paper is recommended for geospatial analysis using retrospective data, mostly missing Eircodes, and for any prospective datasets that do not capture Eircodes to a high level of completeness.
